# Clinical Evaluation of Neonatal Arrhythmias: Experience from a Specialized Pediatric Cardiac Center

**DOI:** 10.3390/jcdd13020065

**Published:** 2026-01-27

**Authors:** Halise Zeynep Genc, Elnur Karimov, Seyma Yakut, Dilek Yavuzcan Ozturk, Demet Oguz, Merih Cetinkaya, Gulhan Tunca Sahin, Erkut Ozturk

**Affiliations:** 1 Department of Pediatric Cardiology, Saglik Bilimleri University Basaksehir Cam and Sakura City Hospital, Istanbul 34480, Turkey; elnurkarimov91@gmail.com (E.K.); gultunca@dr.com (G.T.S.); erkut.ozturk@sbu.edu.tr (E.O.); 2 Department of Pediatrics, Saglik Bilimleri University Basaksehir Cam and Sakura City Hospital, Istanbul 34480, Turkey; yakutseyma@gmail.com; 3 Department of Neonatology, Saglik Bilimleri University Basaksehir Cam and Sakura City Hospital, Istanbul 34480, Turkey; d.yavuzcanozturk@saglik.gov.tr (D.Y.O.); demet.oguz1@saglik.gov.tr (D.O.); merih.cetinkaya@saglik.gov.tr (M.C.)

**Keywords:** neonate, arrhythmia, prenatal diagnosis, antiarrhythmic therapy, supraventricular tachycardia

## Abstract

Neonatal arrhythmias, though relatively uncommon, can range from benign self-limiting conditions to life-threatening disorders requiring intensive management. Data on their clinical spectrum, management, and outcomes remain limited. This study aimed to evaluate the types, frequency, clinical characteristics, treatment strategies, and prognosis of neonatal arrhythmias in a tertiary pediatric cardiac center. We retrospectively reviewed neonates diagnosed with arrhythmia within the first 28 days of life at Basaksehir Cam and Sakura City Hospital between 1 January 2021 and 1 May 2025. Demographic data, electrocardiographic and echocardiographic findings, treatment modalities, recurrence, morbidity, and mortality were analyzed. Patients were categorized as having benign or non-benign arrhythmias. A total of 65 neonates (57% male, mean weight 3.2 kg) were included. Non-benign arrhythmias were more frequent (77%) compared to benign arrhythmias (23%). Supraventricular tachycardia (35%) was the most common non-benign arrhythmia, followed by long QT syndrome (10.7%) and complete atrioventricular block (9.2%). Antiarrhythmic therapy was required in 55% of patients. Pacemaker implantation was performed in seven infants with conduction disorders. Recurrence occurred in 3% of cases, exclusively among patients with supraventricular tachycardia. During a median follow-up of 12.8 months, no mortality was observed. Prenatal diagnosis and early management contribute to favorable outcomes, as reflected in the absence of mortality in this cohort. Larger, prospective studies are warranted to define optimal management strategies and treatment durations for neonatal arrhythmias.

## 1. Introduction

Neonatal arrhythmias (NA), although relatively uncommon, constitute a group of disorders that may lead to severe clinical consequences. The prevalence in the neonatal population has been reported to range from 1% to 5% [1,2]. Neonatal arrhythmias may arise from various systemic and cardiovascular causes. Clinical manifestations vary widely, from asymptomatic presentations to severe cases complicated by congestive heart failure and may occasionally be observed as early as the fetal period [3].

Neonatal arrhythmias can be classified into two categories: benign and non-benign. Benign arrhythmias include premature atrial contractions (PACs), premature ventricular contractions (PVCs), first-degree atrioventricular (AV) block, and junctional rhythms. Non-benign arrhythmias include supraventricular tachycardia (SVT), atrial flutter (AF), ventricular tachycardia (VT), ventricular fibrillation, second- or third-degree AV block, and long QT syndrome [4,5].

Although non-benign arrhythmias are less common than benign arrhythmias, they require early diagnosis and treatment. Although morbidity and mortality rates are higher in this group, the prognosis is generally favorable with appropriate treatment. However, some types of arrhythmias may require long-term antiarrhythmic therapy [6,7,8].

This study aims to evaluate the types, frequency, clinical presentations, risk factors, treatment options, prognosis, recurrence rates, morbidity, and mortality outcomes of neonatal arrhythmias.

## 2. Materials and Methods

This single-center retrospective study was conducted at Basaksehir Cam and Sakura City Hospital between 1 January 2021 and 1 May 2025. The study population included neonates admitted to the neonatal intensive care unit and managed by the Pediatric Cardiology Department, as well as outpatients who presented to the pediatric cardiology clinic, all of whom were diagnosed with arrhythmia within the first 28 days of life. Patients who developed arrhythmias in the postoperative period and those with neurological or metabolic diagnoses were excluded from the study to ensure a homogeneous cohort focusing on primary neonatal arrhythmias and to minimize confounding by transient or reversible rhythm disturbances.

Neonatal arrhythmias were classified as benign and non-benign arrhythmias. Premature atrial contractions (PACs), premature ventricular contractions (PVCs), and first-degree atrioventricular (AV) block were classified as benign arrhythmias. In contrast, PACs were classified as non-benign when they were frequent (>10% over 24 h) and required medical treatment due to the presence of aberrantly conducted or non-conducted beats, associated non-sustained or sustained supraventricular tachycardia, or accompanying clinical signs suggestive of hemodynamic compromise, such as tachypnea, feeding difficulty, or poor feeding. Similarly, PVCs were classified as non-benign when they were frequent (>10% over 24 h) and/or presented as couplets or triplets requiring medical treatment. In neonates, medical therapy was initiated in patients with frequently detected PVCs due to the reported association between frequent ventricular ectopy and the risk of developing cardiomyopathy, and because neonates are unable to reliably report symptoms, which may delay recognition of clinical deterioration. Second- or third-degree AV block and long QT syndrome were also classified as non-benign arrhythmias.

In neonates with complete atrioventricular block and low body weight or prematurity, permanent epicardial pacemaker implantation was postponed until adequate somatic growth was achieved. During this period, patients were managed with temporary pacing wires and closely monitored for hemodynamic stability and signs of heart failure in the pediatric cardiac intensive care unit.

Patients’ 12-lead electrocardiograms (ECGs), 24 h Holter ECG recordings, echocardiograms, presence of prenatal diagnosis, gestational age, birth weight, complaints, genetic investigations, family histories, treatments administered, follow-up periods, and responses to treatment during this period were examined.

Electrocardiography was performed using a Philips PageWriter Trim II device (Philips Medical Systems, Andover, MA, USA) with 12 leads, a speed of 25 mm/s, and an amplitude of 10 mm/mV. Tachycardia was defined as a heart rate at or above the 95th percentile according to age-specific standard values. 12-lead ECG and 24 h Holter ECG recordings were used in the classification of arrhythmias.

The echocardiographic assessment was performed in accordance with the guidelines of the American Society of Echocardiography. All patients were evaluated for the presence of concomitant congenital heart disease. A shortening fraction of <28% or an ejection fraction of <55% was considered an indicator of systolic dysfunction. Patients with a left ventricular end-diastolic diameter z-score > +2 and systolic dysfunction were classified as having dilated cardiomyopathy.

The study was approved by the Basaksehir Cam ve Sakura Ethics Committee on 23 December 2022, under the number 2022.12.413. Informed consent forms were obtained from the parents of all patients included in the study.

### Statistical Analysis

Statistical analyses were performed using IBM SPSS Statistics for Windows, Version 26.0 (IBM Corp., Armonk, NY, USA). The Kolmogorov–Smirnov test was used to assess the normality of continuous variables. Continuous variables are presented as mean ± standard deviation for normally distributed data and as median (interquartile range) for non-normally distributed data. Categorical variables are expressed as frequencies and percentages.

Comparisons between benign and non-benign arrhythmia groups were performed using the independent samples *t*-test or Mann–Whitney U test for continuous variables, as appropriate, and the chi-square test or Fisher’s exact test for categorical variables.

Univariate logistic regression analyses were initially conducted to identify variables potentially associated with arrhythmia type. Variables with a *p* value < 0.20 in univariate analyses were subsequently entered into a multivariable logistic regression model to avoid premature exclusion of clinically relevant predictors in this exploratory cohort. Multicollinearity among covariates was assessed prior to model construction.

Results of logistic regression analyses are presented as odds ratios (ORs) with 95% confidence intervals (CIs). Model fit was evaluated using standard goodness-of-fit measures. A two-sided *p* value <0.05 was considered statistically significant in all analyses.

## 3. Results

Of the total 65 patients included in the study, 37 (57%) were male and 28 (43%) were female. The mean weight of the patients was 3.2 kg (1–5.8). 6 (9.2%) patients were preterm. Non-benign arrhythmias were present in 50 (77%) patients, while benign arrhythmias were present in 15 (23%) patients. The characteristics of the benign and non-benign patient groups were compared (Table 1).

In the univariate analysis, a statistically significant difference was found only in the cardiac disease variable (*p* = 0.01). Logistic regression analysis confirmed that the risk of developing benign arrhythmia in newborns with cardiac disease is significantly higher than that of non-benign arrhythmia. In multivariable logistic regression analysis, the presence of underlying cardiac disease was significantly associated with the occurrence of benign arrhythmias.

**Figure 1:**
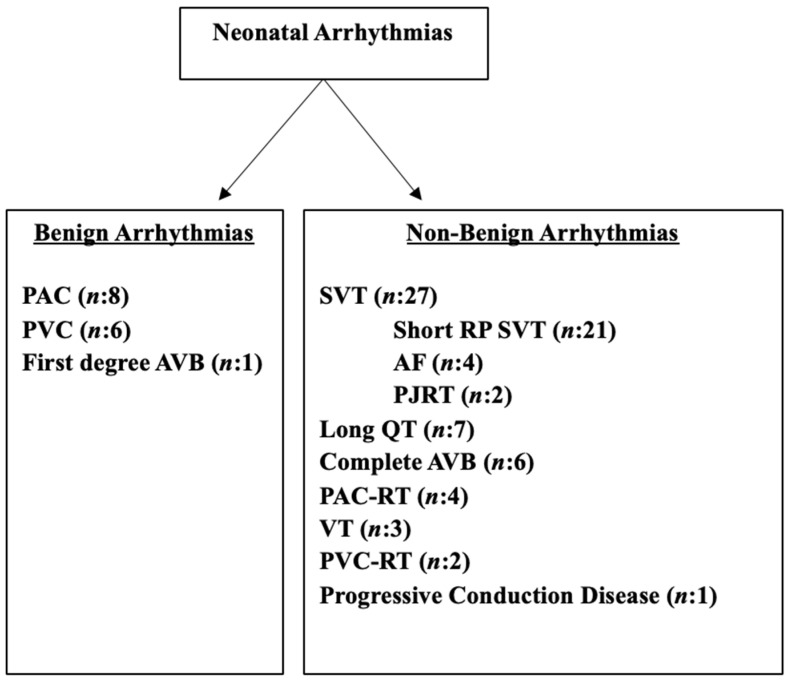
Types of neonatal arrhythmias; AF: atrial flutter, AVB: atrioventricular block, PAC: premature atrial contraction, PAC-RT: premature atrial contraction requiring treatment, PJRT: permanent junctional reciprocal tachycardia, PVC: premature ventricular contraction, PVC-RT: premature ventricular contraction requiring treatment, SVT: supraventricular tachycardia, VT: ventricular tachycardia.

When examined in order of frequency, the most common benign arrhythmias were PAC (12.3%) and PVC (9.2%). First-degree AV block was observed in one patient (1.5%). Among non-benign arrhythmias, supraventricular tachycardia (35%) was the most common, followed by long QT syndrome (10.7%), complete atrioventricular block (9.2%), atrial flutter (6%), premature atrial contractions requiring treatment (6%), ventricular tachycardia (4.6%), ventricular extrasystoles requiring treatment (3%), and progressive cardiac conduction disease (1.5%) (Figure 1).

### 3.1. Tachyarrhythmias

Tachyarrhythmia was detected in 50 of the total 65 patients (77%). Of the patients in the tachyarrhythmia group, 30 (60%) were male and 20 (40%) were female. Fifteen of these patients (30%) had a diagnosis of arrhythmia in the prenatal period.

**Figure 2:**
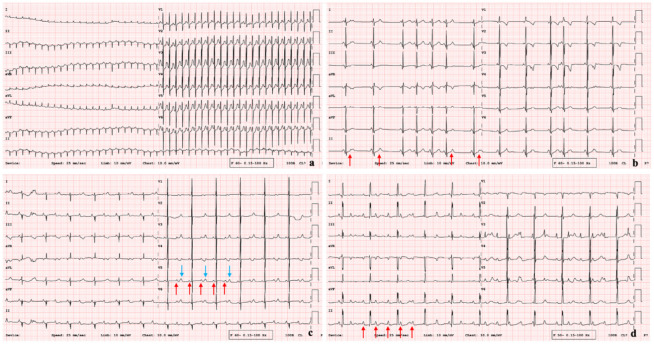
Representative electrocardiographic findings in neonatal arrhythmias, (**a**) supraventricular tachycardia, (**b**) nonconducted premature atrial contractions; red arrows indicate nonconducted P waves, (**c**) long QT syndrome type 3 with functional 2:1 atrioventricular block; red arrows indicate P waves and blue arrows indicate T waves, (**d**) congenital complete atrioventricular block; red arrows indicate P waves.

Twenty-seven of the cases diagnosed with arrhythmia were supraventricular tachycardia (SVT) (Figure 2a). Within this group, permanent junctional reciprocal tachycardia (PJRT) was detected in 2 patients, focal atrial tachycardia and atrial flutter in 4 patients, and short RP SVT in 17 patients (3 of whom had Wolff-Parkinson-White [WPW] syndrome). In addition, four patients had frequent PAC with and/or without aberrant conduction requiring treatment (Figure 2b), and eight patients had low-frequency benign PAC that did not require treatment.

PVC was present in 8 patients. In 2 of these, frequent PVC requiring treatment was identified, while in 6, benign PVC of low frequency that did not require treatment was detected.

Ventricular tachycardia (VT) was identified in a small subset of patients within the tachyarrhythmia group. VT episodes were observed in three patients. One patient had no underlying structural heart disease, while two patients were diagnosed with transposition of the great arteries (TGA). In the patient without structural heart disease, VT was detected during hospitalization in the neonatal intensive care unit for respiratory distress. The arrhythmia demonstrated a right bundle branch block morphology with a superior axis, consistent with left posterior fascicular ventricular tachycardia. The patient had sustained ventricular tachycardia with preserved hemodynamic stability and was successfully converted to sinus rhythm with intravenous amiodarone, followed by oral amiodarone therapy. In both patients with TGA, VT was detected during preoperative monitoring. The arrhythmias exhibited a left bundle branch block morphology with an inferior axis, consistent with a right ventricular outflow tract origin. In one patient, frequent non-sustained VT episodes and frequent premature ventricular contractions (PVC) were initially treated with propranolol; due to persistence of VT, flecainide was subsequently added. In the other patient, frequent non-sustained VT was observed, and sinus rhythm was restored with intravenous amiodarone, followed by maintenance therapy with oral sotalol.

Twenty-five patients received single antiarrhythmic therapy (most commonly propranolol [*n* = 15], followed by propafenone [*n* = 7]). Ten patients received dual antiarrhythmic therapy (propranolol plus amiodarone in 8 patients, propranolol plus flecainide in 2 patients), while two patients received triple antiarrhythmic therapy (propranolol, amiodarone, and flecainide). Fourteen patients were followed without medication, 8 of whom had low-frequency PAC and 6 had low-frequency PVC.

During the follow-up period, recurrence occurred in 2 patients under treatment. In one patient with WPW syndrome, multiple SVT episodes were observed while on propranolol, leading to the addition of amiodarone. After four months without SVT episodes, the patient experienced a recurrence one month following the discontinuation of amiodarone, at which point therapy was switched to sotalol. In another patient with SVT, amiodarone was added due to an SVT episode despite propranolol treatment.

Cardioversion was performed in four patients diagnosed with atrial flutter (AF) on the first postnatal day. None of the infants presented with cardiogenic shock or required cardiopulmonary resuscitation at initial presentation.

The findings for patients with tachyarrhythmia are presented in Table 2.

### 3.2. Bradyarrhythmias

Bradyarrhythmia was identified in 15 patients (23%) included in the study. Of this group, 8 (53%) were female and 7 (47%) were male. Seven patients (47%) were diagnosed prenatally. Subtype analysis revealed long QT syndrome in 7 patients, complete atrioventricular (AV) block in 6 patients, progressive cardiac conduction disease in 1 patient, and first-degree AV block in 1 patient.

A total of seven patients were diagnosed with long QT syndrome. In two patients, the diagnosis was prompted by a positive family history of long QT syndrome (one with affected mother and grandmother, and one with an affected sister). In the remaining patients, prolonged corrected QT (QTc) intervals were identified during evaluation for bradycardia. The QTc intervals ranged from 480 to 544 ms on surface electrocardiography. Genetic testing revealed a pathogenic SCN5A mutation consistent with long QT syndrome type 3, accompanied by 2:1 atrioventricular block, in one patient (Figure 2c); a KCNH2 mutation consistent with long QT syndrome type 2 in one patient; and a KCNQ1 mutation consistent with long QT syndrome type 1 in one patient. Genetic testing was negative in one patient, while three patients were lost to follow-up and therefore could not undergo genetic evaluation.

A total of 7 patients (11%) underwent epicardial pacemaker implantation; 6 of these had congenital complete AV block (Figure 2d) and 1 had progressive cardiac conduction disease. In 2 patients with low birth weight, temporary epicardial pacing wires were placed prior to permanent pacemaker implantation.

Family history analysis revealed that the mother of a patient with complete AV block had Sjögren’s syndrome; the mother and grandmother of a patient with long QT syndrome were also diagnosed with long QT syndrome; and the sister of another patient with long QT syndrome was likewise affected.

Clinical and diagnostic features of the bradyarrhythmia group are summarized in Table 3.

### 3.3. Clinical Presentation

When the diagnostic pathway was evaluated, 29 patients (44.6%) were diagnosed during routine examination due to the detection of arrhythmia or identification of ectopic beats/arrhythmias on ECG or echocardiography. Fourteen patients (21.5%) were diagnosed during NICU hospitalization due to tachycardia or bradycardia. Prenatal arrhythmia was detected in 22 patients (33.8%), of whom five were diagnosed with complete AV block, 2 with bradycardia, and 15 with tachycardia. The mean number of Holter examinations was 2.9 (1–30).

### 3.4. Presence of Congenital Heart Disease

A total of 9 patients (13.8%) had concomitant congenital heart disease. These included transposition of the great arteries (D-TGA, *n*:4), congenitally corrected transposition of the great arteries (C-TGA, *n*:2), total anomalous pulmonary venous drainage (TAPVD, *n*:2), and partial atrioventricular septal defect (pAVSD, *n*:1).

Among the patients with D-TGA, 1 had low-frequency PVC, 1 had frequent PVC, 1 had low-frequency PAC, and 1 had VT. Among the patients with C-TGA, 1 had first-degree AV block and 1 had complete AV block. SVT was observed in a patient with partial AVSD. Atrial flutter was detected in one of the TAPVD cases and SVT in the other.

### 3.5. Genetic Analysis

Genetic testing was performed in patients followed for long QT syndrome. As a result, a heterozygous SCN5A mutation was identified in one patient, and a heterozygous KCNH2 mutation was identified in another. In a patient with prenatal bradycardia who, during postnatal follow-up, exhibited bradycardia, long QT, first- and second-degree type 1 and type 2 AV block, as well as complete right bundle branch block (RBBB), a TRPM4 mutation was detected. This patient was diagnosed with progressive cardiac conduction disease, a condition reported as extremely rare in the literature.

### 3.6. Follow-Up

Among patients with tachyarrhythmias, 33 were followed without medication, while five received propranolol, one received sotalol, and one received flecainide. All patients who required pharmacological management were treated with single-agent antiarrhythmic therapy.

Recurrence was observed in 3% of all patients, both cases diagnosed with SVT. Eight patients received medical therapy for more than one year, including 7 with SVT (2 of whom had WPW syndrome) and 1 with VT. Patients with SVT were treated with propranolol, whereas the patient with VT received propranolol and flecainide. The mean follow-up duration was 12.8 months (1–40). Of the patients with tachyarrhythmia, follow-up data were available for 40 patients, while 10 patients were lost to follow-up and were therefore excluded from recurrence and long-term outcome analyses. Similarly, among patients with bradyarrhythmia, follow-up data were available for 12 patients, whereas 3 patients were lost to follow-up and excluded from recurrence and long-term outcome analyses. No mortality was observed during the follow-up period.

## 4. Discussion

This single-center, retrospective study provides a comprehensive analysis of neonatal arrhythmias followed up at a tertiary cardiac center. In this single-center cohort, neonatal arrhythmias were classified as benign and non-benign, and their relationships with clinical and demographic variables were examined. In our study, non-benign arrhythmias were detected significantly more frequently than benign arrhythmias. Although studies in the literature have focused on non-benign arrhythmias [4,9], the study conducted by Ran et al. observed a higher frequency of benign arrhythmias [10]. It may have been detected this way because they also included sinus arrhythmia as a benign arrhythmia. In the study conducted by Işık et al., the frequency of non-benign arrhythmia was found to be higher than that of benign arrhythmia, and there was no significant difference between them [11]. The detection of non-benign arrhythmia in 77% of 65 patients in our study is consistent with the feature of our center being a tertiary cardiac center with a high number of patients referred.

The most noteworthy finding in our study was the higher rate of congenital cardiac disease in the benign arrhythmia group compared to the non-benign group (40.0% vs. 10.9%; *p* = 0.04). This result seems paradoxical at first glance, given that structural heart disease is generally perceived as a risk factor for serious arrhythmias. However, due to the intensive monitoring and frequent electrocardiography/echocardiography checks of babies with structural anomalies, benign and usually self-limiting premature atrial or ventricular contractions are more easily recognized in these patients. In contrast, non-benign arrhythmias such as SVT, atrioventricular block, or long QT syndrome most often occur with an acute attack and may develop independently of structural heart disease. In addition, some congenital defects may manifest as either resolving or benign ectopia during the neonatal period. Conversely, diseases such as long QT syndrome, due to channelopathy, progress independently of structural heart disease and predominate in the non-benign arrhythmia group. This pattern has also been demonstrated in some neonatal intensive care unit series in the literature, and it has been reported that non-benign arrhythmias can be seen at a high rate in structurally normal hearts [2,4].

The fact that SVT was the most common arrhythmia type in the tachyarrhythmia group is consistent with the literature. Supraventricular tachycardia (SVT) is the most common type of non-benign arrhythmia in the neonatal period, and with early diagnosis and appropriate treatment, the prognosis is generally favorable [12,13]. Gilljam et al. [14] reported that 52% of patients remained recurrence-free during an average follow-up of one year without pharmacological therapy, which is in agreement with our findings. Ran et al. [10] reported a 50% recurrence rate during the one-year follow-up of 40 patients with a diagnosis of SVT. In our study, recurrence was observed in only two patients, and when compared with the literature, the recurrence rate was found to be considerably lower.

According to Lupoglazoff and Denjoy, WPW syndrome is present in 70% of patients diagnosed with SVT under the age of three months [15], while Gilljam et al. reported a prevalence of 34% [14], and Kundak et al. reported a prevalence of 27% [4]. In our study, WPW was identified in 3 out of 29 patients with SVT, corresponding to a frequency of 10%.

Ventricular tachycardia is very rare in neonates and is usually associated with electrolyte disturbances, cardiomyopathy, or congenital heart disease [16]. In our study, consistent with the literature, ventricular tachycardia (VT) was observed in 3 out of 50 patients diagnosed with tachycardia, corresponding to a rate of 6%. One of these patients had concomitant D-transposition of the great arteries (D-TGA).

In our study, no significant association was found between preterm birth, birth weight, sex, prenatal diagnosis, or family history and the type of arrhythmia. Although prenatal diagnosis was observed more frequently in the non-benign arrhythmia group (36.4% vs. 20%), the difference was not statistically significant. It has been reported that the diagnosis of prenatal arrhythmias by fetal echocardiography is being made with increasing frequency, facilitating the early detection and management of non-benign arrhythmias such as SVT or AV block in some series [17,18,19]. Our findings may be attributable to the small sample size, and statistically significant results might be obtained with a larger cohort.

Family history plays a crucial role in inherited channelopathies, especially in cases of long QT syndrome. In our study, although family history appeared to be more frequent in the non-benign group compared with the benign group (14.5% vs. 10%), the difference was not statistically significant (*p* = 1.00). The limited statistical power due to the small sample size, along with the frequent occurrence of novel mutations, may explain why family history demonstrates limited predictive value in the neonatal period.

Complete atrioventricular (AV) block may be associated with congenital heart diseases, most commonly with congenitally corrected transposition of the great arteries (C-TGA). In the presence of a structurally normal heart, maternal rheumatologic disorders such as systemic lupus erythematosus or Sjögren’s syndrome increase the risk of developing atrioventricular block [5,20]. In our study, among six patients diagnosed with complete AV block, one had C-TGA, while another had a maternal history of Sjögren’s syndrome. The mortality associated with complete AV block has been reported to be as high as 20% [21]. In our study, no cases of mortality were observed. We believe that the absence of mortality in patients with complete AV block in our study may be attributed to the fact that all cases were diagnosed prenatally in our center, closely monitored in collaboration with the perinatology clinic, and followed postnatally in our pediatric cardiology intensive care unit.

In neonates diagnosed with SVT, antiarrhythmic medical therapy is administered to reduce the frequency of episodes and to prevent the development of heart failure. The duration of therapy generally ranges from 6 to 12 months but may be individualized according to the clinical condition of the patient. It has been reported that discontinuing therapy in patients with pre-excitation increases the likelihood of recurrence by approximately 2.5 times [22]. In our series, patients received medical therapy for a duration of 6 to 12 months. In addition, in patients with a history of SVT and a diagnosis of WPW syndrome, medical therapy was continued even in the absence of new SVT episodes.

In the literature, the reported mortality rate of neonatal arrhythmias ranges between 6% and 23.6%. In a review evaluating ten studies, 53 deaths were reported among a total of 547 patients with arrhythmias [4,11,14,16,20,23,24,25,26,27]. In our study, no mortality was detected. Similarly, in the study by Doi et al. [9], no mortality was observed, which was attributed to the exclusion of patients with electrolyte disturbances and congenital heart disease from the study population. In our series, however, patients with unrepaired congenital heart disease were also included, and non-benign arrhythmias were observed to be more frequent in this group. While Doi et al. reported a prenatal diagnosis rate of 43.7% [9], our study found rates of 30% in the tachyarrhythmia group and 47% in the bradyarrhythmia group.

The high rate of prenatal diagnosis may be a contributing factor to the low mortality rate.

This study has several limitations. First, its retrospective single-center design and relatively small sample size may limit the generalizability of the findings. Statistically more significant results may be obtained with a larger patient cohort. Second, loss to follow-up in a subset of patients represents an important limitation and may have affected the assessment of recurrence and long-term outcomes. Finally, the observed associations should be interpreted with caution, as referral bias and closer rhythm surveillance in a tertiary cardiac center may have influenced arrhythmia detection, particularly for benign arrhythmias.

## 5. Conclusions

Among neonatal arrhythmias, non-benign types are observed with a noteworthy frequency. Prenatal diagnosis facilitates the early detection of these arrhythmias during the neonatal period and, through timely and appropriate treatment, contributes to the prevention of potential heart failure and mortality. Although the prognosis is generally favorable in neonates receiving appropriate medical therapy, further large-scale studies are needed to determine the optimal duration of treatment. In conclusion, while our findings provide valuable insights into neonatal arrhythmias, further prospective studies with larger patient populations are warranted to more comprehensively evaluate risk factors and elucidate determinants contributing to increased mortality.

## Figures and Tables

**Table 1:** Characteristics of benign and non-benign arrhythmias; CI, confidence interval; MD, mean difference; NA, not applicable; OR, odds ratio.

Variables	Benign (*n* = 15)	Non-Benign (*n* = 50)	OR/MD (95% Cl)	*p* Value
Preterm	0 (0%)	6 (10.9%)	2.76 (0.14−52.82)	0.58
Birth weight (kg)	3.20 ± 1.12	3.21 ± 0.85	−0.01 (NA)	0.99
Male	5 (50%)	32 (58.2%)	1.39 (0.36−5.37)	0.73
Prenatal diagnosis	2 (20%)	20 (36.4%)	2.29 (0.44−11.83)	0.47
Cardiac disease	4 (40%)	6 (10.9%)	0.18 (0.04−0.84)	0.04
Family history	1 (10%)	8 (14.5%)	1.52 (0.17−13.56)	1.0

**Table 2:** Characteristics of tachyarrhythmia patients; AF: atrial flutter, DCC: direct current cardioversion, ECG: electrocardiograph, NA: not available, NICU: neonatal intensive care unit, OC: outpatient clinic, PAC: premature atrial contraction, PAC-RT: premature atrial contraction requiring treatment, PJRT: permanent junctional reciprocal tachycardia, PVC: premature ventricular contraction, PVC-RT: premature ventricular contraction requiring treatment, SVT: supraventricular tachycardia, VT: ventricular tachycardia, WPW: Wolff–Parkinson–White.

Case	Weight	Arrhythmia Type	Prenatal Diagnosis	Gestation Week	Clinical Symptom	Use of DCC	Antiarrhythmic Agents	Treatment Duration (Months)	Follow-Up Period (Months)	Recurrence	Outcomes
1	5.8	PAC	Yes	Term	PN diagnosis	No	No antiarrhythmic treatment	No pharmacological treatment	NA	NA	Lost to follow-up
2	2.6	PAC-RT	No	Term	PAC on ECG, OC	No	Propranolol	6 months	6 months	0	Drug-free follow-up
3	3.8	PAC	No	Term	PAC on ECG, OC	No	No antiarrhythmic treatment	No pharmacological treatment	NA	NA	Lost to follow-up
4	2.3	SVT	No	Term	SVT in NICU	No	Propranolol	6 months	12 months	0	Drug-free follow-up
5	3.4	SVT	Yes	Term	PN diagnosis	No	Propafenone	6 months	NA	NA	Lost to follow-up
6	2.7	PAC-RT	Yes	Term	PN diagnosis	No	Propafenone	1 months	13 months	0	Drug-free follow-up
7	1.5	SVT	No	Preterm	SVT in NICU	No	Propranolol, amiodarone	Amiodarone (5 months), propranolol (9 months)	12 months	0	Drug-free follow-up
8	4	SVT	Yes	Term	SVT on ECG, OC	No	Propranolol, amiodarone	Amiodarone (7 months), propranolol (13 months)	13 months	1	propranolol
9	4.7	SVT	No	Term	SVT in NICU	No	Propranolol	6 months	13 months	0	Drug-free follow-up
10	3	SVT-WPW	No	Term	SVT in NICU	No	Propranolol, amiodarone, sotalol	Amiodarone (5 months), propranolol (5 months), sotalol (8 months)	13 months	2	sotalol
11	3.9	SVT	No	Term	SVT on ECG, OC	No	Propranolol, amiodarone	Amiodarone (4 months) propranolol (8 months)	15 months	0	Drug-free follow-up
12	3	SVT	No	Term	PAC on ECG, OC	No	Propafenone	2 months	17 months	0	Drug-free follow-up
13	2.5	PAC	Yes	Term	PN diagnosis	No	No antiarrhythmic treatment	No pharmacological treatment	22 months	0	Drug-free follow-up
14	2.2	SVT	No	Term	SVT on ECG, OC	No	Propranolol, flecainide	8 months	NA	NA	Lost to follow-up
15	4	SVT	No	Term	SVT on ECG, OC	No	Propranolol, amiodarone	Amiodarone (12 months), propranolol (15 months)	22 months	0	Drug-free follow-up
16	5	SVT	No	Term	SVT on ECG, OC	No	Propafenone	5 months	26 months	0	Drug-free follow-up
17	3	AF	No	Term	SVT in NICU	Yes	Propranolol	10 months	24 months	0	Drug-free follow-up
18	4.1	PAC	No	Term	PAC on ECG, OC	No	No antiarrhythmic treatment	No pharmacological treatment	20 months	0	Drug-free follow-up
19	4.2	PAC	No	Term	PAC on ECG, OC	No	No antiarrhythmic treatment	No pharmacological treatment	30 months	0	Drug-free follow-up
20	2.2	PAC-RT	Yes	Term	PN diagnosis	No	Propranolol	6 months	30 months	0	Drug-free follow-up
21	3	PAC	No	Preterm	PAC on ECG, OC	No	No antiarrhythmic treatment	No pharmacological treatment	18 months	0	Drug-free follow-up
22	2.1	SVT-PJRT	Yes	Preterm	PN diagnosis	No	Propranolol, amiodarone	Amiodarone (4 months), propranolol (6 months)	19 months	0	Drug-free follow-up
23	3.1	SVT-WPW	No	Term	SVT on ECG, OC	No	Propranolol	28 months	28 months	0	propranolol
24	3.4	SVT	No	Term	PAC on ECG, OC	No	Propafenone	6 months	28 months	0	Drug-free follow-up
25	3.3	SVT	No	Term	SVT in NICU	No	Propranolol	9 months	24 months	0	Drug-free follow-up
26	2.7	AF	Yes	Term	PN diagnosis	Yes	Propranolol, amiodarone	Amiodarone (3 months), propranolol (6 months)	24 months	0	Drug-free follow-up
27	3	SVT	Yes	Term	PN diagnosis	No	Propafenone	6 months	32 months	0	Drug-free follow-up
28	3.7	PAC	No	Term	PAC on ECG, OC	No	No antiarrhythmic treatment	No pharmacological treatment	24 months	0	Drug-free follow-up
29	3.4	SVT	Yes	Term	PN diagnosis	No	Propranolol	30 months	30 months	0	propranolol
30	3.5	SVT-PJRT	Yes	Term	PN diagnosis	No	Propranolol, amiodarone, flecainide	Amiodarone (5 months), propranolol (9 months), flecainide (7 months)	32 months	0	Drug-free follow-up
31	4.6	SVT	No	Term	PAC on ECG, OC	No	Propranolol	24 months	30 months	0	Drug-free follow-up
32	2.5	AF	No	Term	SVT in NICU	Yes	Propranolol, amiodarone, flecainide	Amiodarone (3 months), propranolol (6 months), flecainide (1 months)	42 months	0	Drug-free follow-up
33	3.8	SVT-WPW	No	Term	SVT in NICU	No	Propranolol	42 months	42 months	0	Propranolol
34	3.4	SVT	No	Term	SVT in NICU	No	Propranolol, amiodarone	Amiodarone (5 months), propranolol (24 months)	40 months	0	Drug-free follow-up
35	3.1	PAC-RT	Yes	Term	PN diagnosis	No	Propranolol	NA	NA	NA	Lost to follow-up
36	3.2	AF	Yes	Term	PN diagnosis	Yes	Propranolol	NA	NA	NA	Lost to follow-up
37	3.4	SVT	Yes	Term	PN diagnosis	No	Propafenone	NA	NA	NA	Lost to follow-up
38	3	PAC	No	Term	PAC in NICU	No	No antiarrhythmic treatment	No pharmacological treatment	NA	NA	Lost to follow-up
39	3	SVT	No	Term	SVT in NICU	No	Propranolol	7 months	9 months	0	Drug-free follow-up
40	1.5	PVC-RT	No	Preterm	PVC in NICU	No	Propranolol	2 months	2 months	0	Propranolol
41	2.5	PVC	No	Term	PVC in NICU	No	No antiarrhythmic treatment	No pharmacological treatment	2 months	0	Drug-free follow-up
42	2	PVC	Yes	Term	PN diagnosis	No	No antiarrhythmic treatment	No pharmacological treatment	8 months	0	Drug-free follow-up
43	4	PVC-RT	No	Term	Seizure	No	Propranolol	1 months	10 months	0	Drug-free follow-up
44	4	PVC	No	Term	PVC on ECG, OC	No	No antiarrhythmic treatment	No pharmacological treatment	9 months	0	Drug-free follow-up
45	3.8	VT	No	Term	VT in NICU	No	Propranolol, flecainide	Propranolol (12 months), flecainide (18 months)	18 months	0	Flecainide
46	3	PVC	No	Term	PVC on ECG, OC	No	No antiarrhythmic treatment	No pharmacological treatment	24 months	0	Drug-free follow-up
47	3.3	VT	No	Term	VT in NICU	No	Amiodarone	NA	NA	NA	Lost to follow-up
48	4.6	PVC	No	Term	PVC on ECG, OC	No	No antiarrhythmic treatment	No pharmacological treatment	6 months	0	Drug-free follow-up
49	4.4	PVC	No	Term	PVC on ECG, OC	No	No antiarrhythmic treatment	No pharmacological treatment	NA	NA	Lost to follow-up
50	3.4	VT	No	Term	VT in NICU	No	Sotalol	2 months	9 months	0	Drug-free follow-up

**Table 3:** Characteristics of bradyarrhythmia patients; C-TGA: corrected transposition of the great arteries, NA: Not Available, WPW: Wolff–Parkinson–White.

Case	Weight (kg)	Arrhythmia Type	Prenatal Diagnosis	Gestation Week	PMI	Indication for PMI	Treatment	Follow-Up Period (Months)	Genetic Mutation	Family History/ Congenital Heart Disease
1	3.2	Complete AV block	Yes	Term	Yes	Low heart rate	Pacemaker implantation	9 months	NA	None
2	3	Complete AV block	Yes	Term	Yes	Low heart rate	Pacemaker implantation	10 months	NA	Sjögren’s disease in the mother
3	3	Long QT syndrome	No	Term	No	Not indicated	Propranolol	NA	NA	None
4	3.2	Complete AV block	Yes	Term	Yes	Low heart rate	Pacemaker implantation	13 months	NA	None
5	1.6	Complete AV block	Yes	Preterm	Yes	Low heart rate	Pacemaker implantation	13 months	NA	None
6	1.5	Long QT syndrome	Yes	Preterm	No	Not indicated	Propranolol	5 months	KCNQ1	WPW in the mother
7	1.5	Complete AV block	No	Preterm	Yes	Low heart rate	Pacemaker implantation	36 months	NA	None
8	3.8	Long QT syndrome	No	Term	No	Not indicated	Propranolol, mexiletine	15 months	SCN5A	Long QT syndrome in the mother and grandmother
9	4	Long QT syndrome	No	Term	No	Not indicated	Propranolol	15 months	KCNH2	Long QT syndrome in sister
10	2.7	Long QT syndrome	No	Term	No	Not indicated	Propranolol	33 months	NA	None
11	3.8	First degree AV block	No	Term	No	Not indicated	No treatment required	38 months	NA	C-TGA in a patient
12	3	Progressive cardiac conduction disease	Yes	Term	Yes	Low heart rate	Pacemaker implantation	40 months	TRPM4	None
13	3	Long QT syndrome	No	Term	No	Not indicated	Propranolol	NA	NA	None
14	2.7	Long QT syndrome	No	Term	No	Not indicated	Propranolol	NA	NA	None
15	3.2	Complete AV block	Yes	Term	Yes	Low heart rate	Pacemaker implantation	4 months	NA	C-TGA in a patient

## Data Availability

The data presented in this study are available on request from the corresponding author.
